# Nanometa Live: a user-friendly application for real-time metagenomic data analysis and pathogen identification

**DOI:** 10.1093/bioinformatics/btae108

**Published:** 2024-02-24

**Authors:** Kristofer Sandås, Jacob Lewerentz, Edvin Karlsson, Linda Karlsson, David Sundell, Kotryna Simonyté-Sjödin, Andreas Sjödin

**Affiliations:** Division of CBRN Defence and Security, Swedish Defence Research Agency (FOI), Umeå 906 21, Sweden; Division of CBRN Defence and Security, Swedish Defence Research Agency (FOI), Umeå 906 21, Sweden; Division of CBRN Defence and Security, Swedish Defence Research Agency (FOI), Umeå 906 21, Sweden; Division of CBRN Defence and Security, Swedish Defence Research Agency (FOI), Umeå 906 21, Sweden; Division of CBRN Defence and Security, Swedish Defence Research Agency (FOI), Umeå 906 21, Sweden; Pediatrics, Department of Clinical Sciences, Umeå University, Umeå 901 87, Sweden; Division of CBRN Defence and Security, Swedish Defence Research Agency (FOI), Umeå 906 21, Sweden

## Abstract

**Summary:**

Nanometa Live presents a user-friendly interface designed for real-time metagenomic data analysis and pathogen identification utilizing Oxford Nanopore Technologies’ MinION and Flongle flow cells. It offers an efficient workflow and graphical interface for the visualization and interpretation of metagenomic data as it is being generated. Key features include automated BLAST validation, streamlined handling of custom Kraken2 databases, and a simplified graphical user interface for enhanced user experience. Nanometa Live is particularly notable for its capability to run without constant internet or server access once installed, setting it apart from similar tools. It provides a comprehensive view of taxonomic composition and facilitates the detection of user-defined pathogens or other species of interest, catering to both researchers and clinicians.

**Availability and implementation:**

Nanometa Live has been implemented as a local web application using the Dash framework with Snakemake handling the data processing. The source code is freely accessible on the GitHub repository at https://github.com/FOI-Bioinformatics/nanometa_live and it is easily installable using Bioconda. It includes containerization support via Docker and Singularity, ensuring ease of use, reproducibility, and portability.

## 1 Introduction

Metagenomics is a relatively young field that has made significant contributions to various areas, including bacterial taxonomy, viral ecology, and medical diagnostics (Quince *et al.* 2017). The efficiency and affordability of second- and third-generation sequencing technologies have led to the generation of vast amounts of data in metagenomics (Loman and Pallen 2015). This abundance of data necessitates the development of accessible analysis and interpretation methods that can cater to a wide audience. Metagenomics provides a rapid, sensitive, and species nonspecific alternative to traditional pathogen detection methods such as rapid tests or polymerase chain reaction (Lindner and Renard 2013). Moreover, third-generation sequencers such as the Oxford Nanopore can deliver data in real-time, enabling faster analysis, which is crucial in epidemiological investigation of disease outbreaks (Quick *et al.* 2015).

Several tools currently exist for real-time visualization of metagenomic data, including WIMP (Juul *et al.* 2015), MARTi (https://github.com/richardmleggett/MARTi), MAIRA (Albrecht *et al.* 2020), and CRuMPIT (Sanderson *et al.* 2018). Other tools focus on pathogen detection, such as MetaPORE (Greninger *et al.* 2015), while some provide interactive overviews of species, like Pavian (Breitwieser and Salzberg 2020) and Krona (Ondov *et al.* 2011). However, there is currently no single application that offers general species classification, real-time pathogen detection, the ability to operate without internet access during sequencing, and the use of custom databases. Nanometa Live fills this niche by integrating these key features into both its workflow and a graphical user interface (GUI).

## 2 Materials and methods

**Figure 1.**
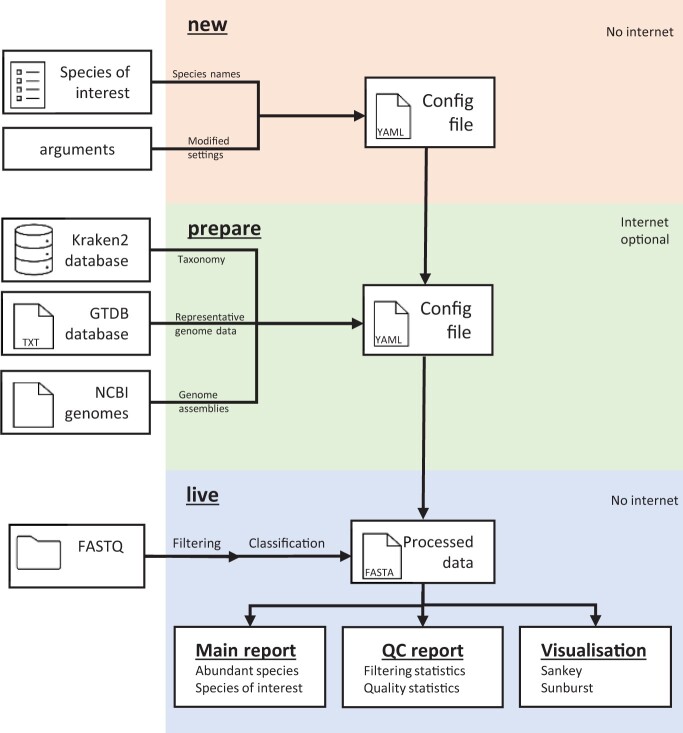
Nanometa Live Workflow. This flowchart depicts the key stages of Nanometa Live, from initial data configuration to real-time analysis in the GUI, highlighting the streamlined process for transforming FASTQ files into dynamic visualizations and reports.

Nanometa Live combines a sophisticated backend workflow with an interactive frontend GUI, enabling comprehensive real-time analysis of metagenomic data (Fig. 1). This system is adept at processing FASTQ files generated on-the-fly by Oxford Nanopore Technologies instruments.

The process begins with the nanometa-new command, which allows users to efficiently update the configuration file using optional arguments. This step tailors the workflow according to specific research needs, including species of interest and analysis parameters. Subsequently, the nanometa-prepare command is executed, focusing on essential preparatory tasks. This includes downloading reference genomes, extracting taxonomic IDs from the Kraken2 database, and building BLAST databases. Notably, this process allows for optional internet use; while automated downloading is available with internet access, users can also manually provide files in offline settings.

The nanometa-live command is the core of Nanometa Live, controlling both the Snakemake (Mölder *et al.* 2021) backend processing and the frontend GUI. As sequencing progresses, new FASTQ file batches are seamlessly integrated into the workflow for real-time analysis. This includes initial filtering using FastP (Chen *et al.* 2018), followed by classification with Kraken2 (Wood *et al.* 2019, 2). Quality control data, such as read counts and base pairs sequenced, are extracted and processed for each batch. Sequences that align with user-defined species of interest are parsed using KrakenTools (Lu *et al.* 2022) and validated through BLAST (Altschul *et al.* 1990) searches against the selected reference genomes.

Developed with Dash and Plotly frameworks, Nanometa Live’s GUI is a dynamic web application that updates with the latest backend results. It offers user-friendly data visualization and exploration, with direct saving of plots and intuitive displays for taxon abundance and other important metrics.

## 3 Features

Nanometa Live’s GUI features live updates that can be paused, allowing for detailed exploration and filtration of data at any stage of sequencing. When updates are resumed, the GUI dynamically refreshes with the latest results from the workflow. The interface is intuitively divided into three main tabs: Main, QC, and two additional visualization tabs: Sankey and Sunburst.

In the Main tab, the focus is on providing immediate insights into critical data. This is achieved through two comprehensive tables: “Most Abundant Species” and “Species of Interest”. These tables present a detailed, filterable view of the sample’s taxonomic composition, enabling users to swiftly pinpoint the most prevalent taxa and those of particular interest to their study.

The QC tab is dedicated to essential quality control metrics of the sequencing process. It displays a comparative count of reads that have been successfully classified against those that haven’t. Additionally, the tab keeps track of batch files awaiting processing. Enhancing the user’s understanding of sequencing performance and throughput, bar charts visually represent the cumulative and noncumulative counts of reads and base pairs over time.

The Sankey tab in Nanometa Live features an interactive Sankey diagram, purposefully designed to visually represent the distribution of sequencing reads across taxonomic levels. This diagram simplifies the understanding of the hierarchical structure within the data, highlighting the flow and relative abundance of different taxa. It serves as an intuitive approach to grasping the major lineages and their proportional representation in the sample. Complementing this, the Sunburst presents a plot, facilitating an exploration of the sample’s taxonomic composition. This plot employs a radial layout where each concentric ring represents a different taxonomic level, providing users with a comprehensive view of the data’s hierarchical complexity. This visualization is particularly effective for a granular examination of taxonomic distribution and for contrasting the relative abundances at various hierarchical levels.

## 4 Conclusion

Nanometa Live provides an platform installable through Bioconda (Grüning *et al.* 2018) for real-time metagenomic data analysis and pathogen identification. A key feature is its ability to operate independently without the need for continuous internet access post-installation, an essential advantage in various research and clinical settings.

The tool’s interactive GUI is specifically designed for a comprehensive and intuitive presentation of sample taxonomic composition. Enhancements include support for custom Kraken2 databases, enabling users to adapt the tool to specific research requirements and unique microbial communities, thus broadening its applicability in diverse metagenomic studies.

With its user-friendly visualization capabilities, Nanometa Live is accessible to a wide spectrum of users, from researchers to clinicians, regardless of their bioinformatics expertise. In summary, Nanometa Live combines offline functionality and customizable database support in a user-centered design, marking it as a vital instrument for real-time metagenomic analysis across various environments.

## Data Availability

The tutorial data underlying this article are available in Figshare, at https://doi.org/10.6084/m9.figshare.24233020.v1.
